# A234 IN VITRO GUT MICROBIOME AND METABOLITE RESPONSES TO RESISTANT STARCH ARE INDIVIDUALIZED

**DOI:** 10.1093/jcag/gwab049.233

**Published:** 2022-02-21

**Authors:** P Dobranowski, H Qin, K Walker, J Butcher, A Gowing, R Singleton, J Mayne, D R Mack, D Figeys, A Stintzi

**Affiliations:** 1 Biochemistry, Microbiology and Immunology, University of Ottawa Faculty of Medicine, Surrey, BC, Canada; 2 Children’s Hospital of Eastern Ontario, Ottawa, ON, Canada

## Abstract

**Background:**

Gut microbes degrade and ferment resistant starch (RS) into metabolites that help maintain gut homeostasis. Clinical trials have evaluated RS for various health conditions, but individuals respond to RS with profound variability. The reason for this variability is unclear.

**Aims:**

Using *in vitro* culturing methods and multi-omic analyses, we hypothesize that individuals will elicit variable responses to RS with respect to overall fermentation, bacterial composition, short chain acid production, and metabolite flux.

**Methods:**

As part of an ongoing clinical trial, we have selected 4 pediatric patients with inflammatory bowel disease to better understand microbiome-RS interactions. We cultured stools anaerobically using a high-throughput platform (“RapidAIM”) with 9 different pre-digested RS. After 18-hour incubations, media supernatants were used to measure pH and perform targeted and semi-targeted metabolomic analyses with a panel of 116 compounds. Bacterial pellets were isolated for 16S rRNA gene sequencing analyses to evaluate changes in microbiome compositions. Data were analyzed with generalized linear mixed models, principal component analysis (PCA), random forest (RF) classification with feature selection, and network construction with graphical lasso.

**Results:**

Changes in several microbiome parameters were different across individuals, including the magnitude of pH changes, metabolite signatures, and relative abundances of important bacterial taxa. Bacterial species known to degrade RS were more abundant in individuals showing stronger RS fermentation. Inter-individual discrimination was accomplished with PCA and RF, from which we could identify metabolite signatures. The robustness of microbiome networks corresponded to RS fermentation and butyrate production.

**Conclusions:**

We report a novel perspective on how individuals respond to RS’ differently. Butyrate remains an important hub of the microbiome architecture with respect to RS fermentation. Future work will interrogate the roles of individualized metabolomic responses on host physiology. *In vivo* responses to RS are being evaluated in an ongoing clinical trial.

**Funding Agencies:**

CCC, CIHRGenome Ontario, Genome Canada

